# Supplementary material for: Prevalence of Intestinal Parasitic Infections, Genotypes, and Drug Susceptibility of Giardia lamblia among Preschool and School-Aged Children: A Cross-Sectional Study in Thailand
Source: Trop Med Infect Dis. 2023 Aug 1;8(8):394. doi: 10.3390/tropicalmed8080394 (PMC10457730; doi:10.3390/tropicalmed8080394)

Table S1: Probes and primers used for the screening and genotyping of Giardia from stool samples

	Target gene	Sequence	Reference (GenBank Accession No.)
Probe: GD-P	SSU rRNA	VIC-CCCGCGGCGGTCCCTGCTAG-MGB	M54878
Primer: GD-F		GACGGCTCAGGACAACGGTT	Liu et al., 2013
Primer: GD-P		TTGCCAGCGGTGTCCG	Llewellyn S., et al. (2016)
Probe: GiardA-P	TPI A	FAM-CGCTGCTATCCTCAACTG-MGB	XM_001706778 Modified (Almeida et al., 2010)
Primer: GiardA-F		CGTACACCTGTCAACAGCCATT	
Primer: GiardA-R		CCTGAAGCATCTCAACACTTGTCT	
Probe: GiardB-P	TPI B	VIC-ATTCTCCAATCTCCTTCTTAAG-MGB	KP687784 Modified (Almeida et al., 2010)
Primer: GiardB-F		GATGAACGCAAGGCCAATAA	
Primer: GiardB-R		GGCAATTACAACGTTCTCCCATA	

Table S2: Frequency of symptoms in study participants

Symptoms & Signs	*Giardia lamblia* infection (N=115)	Non-*Giardia lamblia* infection (N=546)	P-value	Pathogenic (N=131)	Non-pathogenic (N=530)	P-value
Abdominal cramping (%)						
- Having	12 (10.43)	52 (9.52)	0.764	13 (9.92)	51 (9.62)	0.917
- Not having	103 (89.57)	494 (90.48)		118 (90.08)	479 (90.38)	
Nausea						
- Having	2 (1.74)	8 (1.47)	0.827	2 (1.53)	8 (1.51)	0.988
- Not having	113 (98.26)	538 (98.53)		129 (98.47)	522 (98.49)	
Vomiting						
- Having	7 (6.09)	23 (4.21)	0.380	7 (5.34)	23 (4.34)	0.621
- Not having	108 (93.91)	523 (95.79)		124 (94.66)	507 (95.66)	
Diarrhea						
- Having	5 (4.35)	22 (4.03)	0.875	6 (4.58)	21 (3.96)	0.749
- Not having	110 (95.65)	524 (95.97)		125 (95.42)	509 (96.04)	
Fever						
- Having	4 (3.48)	32 (5.86)	0.306	5 (3.82)	31 (5.85)	0.359
- Not having	111 (96.52)	514 (94.14)		126 (96.18)	499 (94.15)	
RBCs in stool						
- Having	0 (0)	0 (0)	-	0 (0)	0 (0)	-
- Not having	115 (100)	546 (100)		131 (100)	530 (100)	
WBCs in stool						
- Having	3 (2.61)	6 (1.10)	0.204	3 (2.29)	6 (1.13)	0.306
- Not having	112 (97.39)	540 (98.90)		128 (97.71)	524 (98.87)	

Figure S1. Growth phase of *G. lamblia* WB-C6 strain. Data are expressed as mean± SD of three independent experiments. Correlation factor (r^2) was calculated using linear regression by GraphPad Prism 7.01.

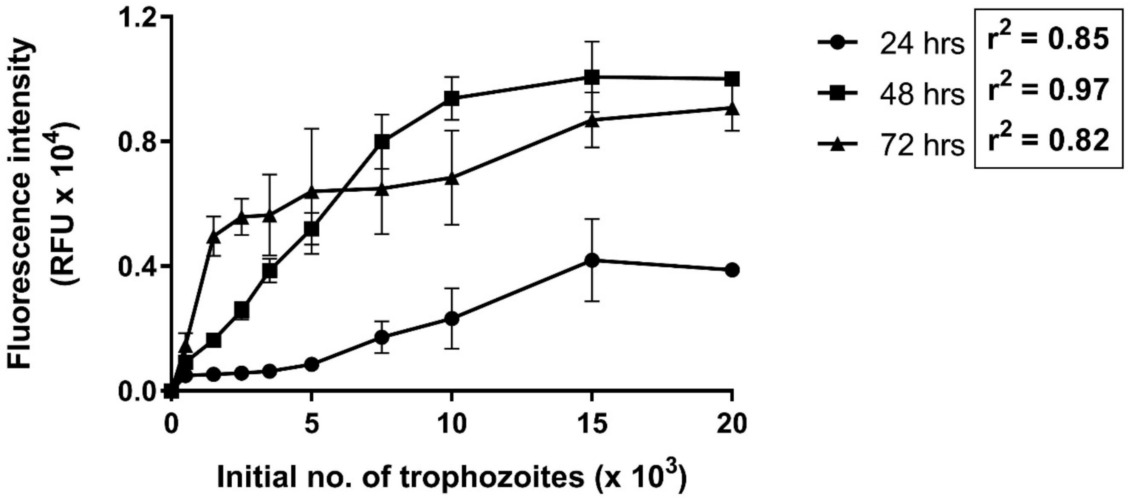

Figure S2. Photomicrographs of excystation and axenization of *G. lamblia* isolate in TYI-S-33 medium. (A) purified cysts before excystation; (B) mobile trophozoites after excystation; (C) trophozoites in monolayer. All images are at 200X magnification.

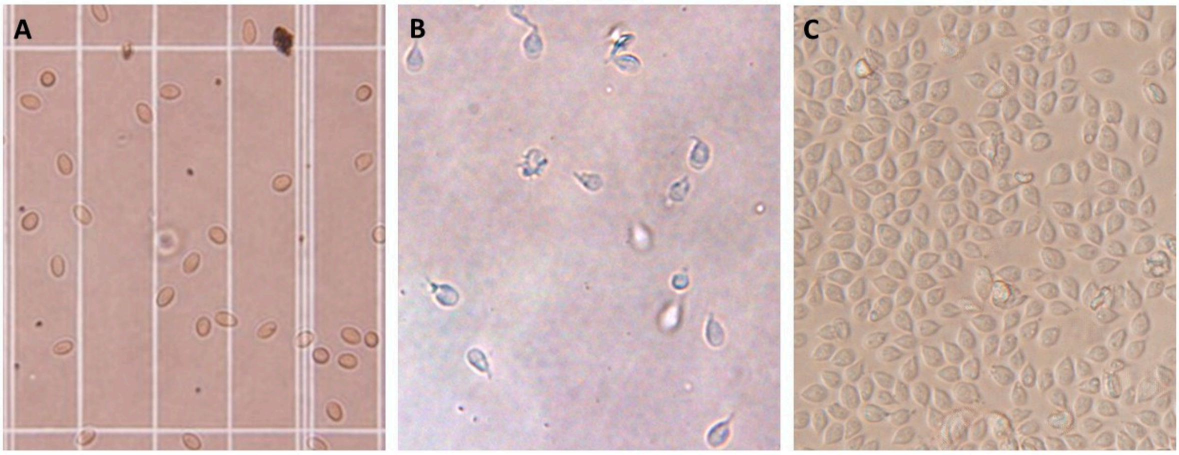

Supplement: Supplementary file 1 [file tropicalmed-08-00394-s001.zip › tropicalmed-2385231-supplementary.pdf]
